# BrownieAligner: accurate alignment of Illumina sequencing data to de Bruijn graphs

**DOI:** 10.1186/s12859-018-2319-7

**Published:** 2018-09-04

**Authors:** Mahdi Heydari, Giles Miclotte, Yves Van de Peer, Jan Fostier

**Affiliations:** 1 Department of Information Technology, Ghent University-imec, IDLab, Ghent, B-9052 Belgium; 2 Bioinformatics Institute Ghent, Ghent, B-9052 Belgium; 3 Center for Plant Systems Biology, VIB, Ghent, B-9052 Belgium; 4 Department of Plant Biotechnology and Bioinformatics, Ghent University, Ghent, B-9052 Belgium; 5 Department of Genetics, Genome Research Institute, University of Pretoria, Pretoria, South Africa

**Keywords:** Next-generation sequencing, Graph alignment, Illumina, de Bruijn Graph, Markov Model

## Abstract

**Background:**

Aligning short reads to a reference genome is an important task in many genome analysis pipelines. This task is computationally more complex when the reference genome is provided in the form of a de Bruijn graph instead of a linear sequence string.

**Results:**

We present a branch and bound alignment algorithm that uses the seed-and-extend paradigm to accurately align short Illumina reads to a graph. Given a seed, the algorithm greedily explores all branches of the tree until the optimal alignment path is found. To reduce the search space we compute upper bounds to the alignment score for each branch and discard the branch if it cannot improve the best solution found so far. Additionally, by using a two-pass alignment strategy and a higher-order Markov model, paths in the de Bruijn graph that do not represent a subsequence in the original reference genome are discarded from the search procedure.

**Conclusions:**

BrownieAligner is applied to both synthetic and real datasets. It generally outperforms other state-of-the-art tools in terms of accuracy, while having similar runtime and memory requirements. Our results show that using the higher-order Markov model in BrownieAligner improves the accuracy, while the branch and bound algorithm reduces runtime. BrownieAligner is written in standard C++11 and released under GPL license. BrownieAligner relies on multithreading to take advantage of multi-core/multi-CPU systems. The source code is available at: https://github.com/biointec/browniealigner

**Electronic supplementary material:**

The online version of this article (10.1186/s12859-018-2319-7) contains supplementary material, which is available to authorized users.

## Background

Modern Illumina machines produce sequencing data with a high throughput at a low financial cost. Reads generated by this platform are relatively short (100-300 bp) and have a relatively low error rate (1-2% errors) [1]. A key data structure to represent and manipulate these data in many bioinformatics applications is the de Bruijn graph. It has been used in different contexts, ranging from *de novo* genome assembly [2], transcriptome assembly [3], metagenomics [4], variant calling and structural variation detection [5].

The de Bruijn graph is a directed graph where nodes correspond to *k*-mers and edges represent an overlap of *k*−1 nucleotides between nodes. When the de Bruijn graph is built from sequencing data and all *k*-mers and their overlaps are present in the input data, the original sequence can be found as some path through the graph. The de Bruijn graph can thus be seen as a compact multiple sequence alignment representation of the input reads.

Aligning reads to a reference genome is a prerequisite step in many genome analysis pipelines. The vast majority of read alignment software aligns short reads to a linear reference genome [6, 7]. A common strategy in these aligners is a “seed-and-extend” paradigm. First, seeds such as maximal exact matches between a read and the reference sequence are identified. Those seeds indicate candidate positions in the reference genome from which the read originated. In the second step, each seed is extended to the left and right until a full read alignment is obtained and the alignments with statistically significant similarity are reported [8].

For certain applications, the reference genome may be provided as a de Bruijn graph rather than a linear sequence. For example in the scaffolding phase of a short read assembler, reads can be aligned to the assembly graph [9]. Additionally, for genome identification of reads with an unknown origin in a metagenomics study, reads can be aligned to a de Bruijn graph that is built from multiple genomes. Recently, two standalone tools have been proposed to align short Illumina reads to de Bruijn graphs: BGREAT [10] and deBGA [11].

In order to align reads to a graph representation of the reference genome, the same seed-and-extend approach can be used. While the seeding phase is straightforward, the extension phase is computationally more expensive when dealing with graphs. Given a seed, a brute-force approach would be an exhaustive search in the graph (e.g. depth-first search (DFS) or breadth-first search (BFS)), exploring all possible branches of the tree until the best alignment is found that covers the entire read. However, the number of visited nodes can grow exponentially in the length of the read. Assuming a four-letter DNA alphabet, each node has up to four outgoing arcs. Therefore, to align a read of size *l*, up to 4^*l*−*k*^ nodes need to be explored in the worst-case scenario. While most of the reads never reach this upper bound, it shows that aligning reads to the graph can potentially be intractable, especially in repetitive regions where the graph contains many branches. To tackle this problem, BGREAT and deBGA have an early stop mechanism, which stops exploring nodes when the number of mismatches exceeds a certain threshold. This strategy reduces the search space but potentially fails to return the optimal solution.

A second complication that arises when aligning reads to a de Bruijn graph is that paths in the graph do not necessarily correspond to a substring of the reference genome. Although two connected nodes in the de Bruijn graph always correspond to two consecutive *k*-mers in the reference genome, paths of three or more connected nodes do not necessarily correspond to a chain of *k*-mers in the reference genome. Therefore, aligning reads to such paths would reduce the overall accuracy of the alignment procedure.

In this paper, we introduce BrownieAligner to align short Illumina reads to a de Bruijn graph. Even though for most practical applications, a de Bruijn graph would be constructed from sequencing data, we assume in this paper that it is built from a known reference genome, thus yielding a complete and error-free de Bruijn graph. This allows us to focus on the accuracy of the actual alignment algorithms unimpeded by superimposed noise from the graph structure itself. For read alignment, the seed-and-extend paradigm is used. We propose additional strategies to narrow down the search space and avoid the alignment to paths in the graph that do not correspond to sequences in the reference genome. First, the exhaustive DFS is augmented with a branch and bound algorithm. For each branch, an upper bound is computed to the alignment score that could be obtained in that branch. The branch is discarded from the search procedure if it cannot improve the best solution found so far. In order to rapidly find candidate solutions with a high score, the DFS greedily prioritizes towards the node that appears best. Secondly, we propose to annotate the graph with information about the paths that do exist in the reference data. This is modeled as a higher-order Markov model (MM). A priori, this information is not present in the de Bruijn graph. We thus propose to perform the alignment in two passes: one alignment pass to train the MM, and a second alignment pass that is guided by the MM to obtain the final alignments. Using this MM improves the overall alignment accuracy. This procedure is similar to the strategy used in STAR to perform spliced alignment of RNA-seq reads: in a first alignment round the aligners learns new splice sites; in the second round, the final alignments are obtained [12].

## Methods

### Read alignment algorithm

In our de Bruijn graph representation, linear paths of connected *k*-mers are contracted to unitigs. Nodes thus represent sequences of length *k* or larger. The problem of finding an optimal read alignment in a graph can be formalized as finding the optimal walk in that graph. A walk is an alternating list *v*_0_,*e*_1_,*v*_1_,…,*e*_*w*_,*v*_*w*_ of nodes and edges such that, for 1≤*i*≤*w*, edge *e*_*i*_ has endpoints *v*_*i*−1_ and *v*_*i*_. In a de Bruijn graph there is at most one edge between two nodes. Therefore the walk can unambiguously be represented as a chain of nodes *v*_0_,*v*_1_,…,*v*_*w*_. It has been shown that given a de Bruijn graph *G*, a read *r* and a cost function *f*, finding an optimal chain in *G* for *r* that minimizes the cost function is an NP-complete problem [10].

Given a read, the first step of finding such optimal chain is finding at least one node of that chain (seeding). Then, by traversing the graph to the left and right, we can find a chain that maximizes a well-defined objective function (extension). BrownieAligner attempts to maximize the similarity score as used in the Needleman-Wunsch algorithm [13]. Therefore a chain that has the highest similarity score to the input read is assumed to be the optimal chain. The advantage of this approach is that it can deal with both substitution errors as well as insertions and deletions. In contrast, the Hamming distance, which is for example used in BGREAT, can only deal with substitutions. In the following, the similarity score of a chain to a given read is defined as the similarity score of the sequence represented by that chain to the read.

BrownieAligner first generates a hash table index of the graph’s *k*-mers (default: *k*=31) to accelerate the seed-finding procedure. Given an input read as a query, it iterates over all *k*-mers of that read and returns a seed for all *k*-mers that exist in the graph. Seeds that are contiguous in both the read and the graph are merged and sorted according to length.

Depending on the *k*-mer size, read length and the error distribution, it is possible that no exact *k*-mer seeds can be found in some reads. In that case, maximal exact matches (MEMs) between the read and the unitigs of the graph are found using the essaMEM library [14]. Those MEMs are necessarily shorter than *k* nucleotides.

The extension phase is straightforward when the entire read is contained within a single unitig. In this case, the seed can be extended to the left and to the right within a single node and the alignment score is easily obtained. However, it is also possible that extending the seed moves the alignment across an edge, into an adjacent unitig. In this case, the aligner should decide at each branching point along which nodes to continue.

Our graph alignment algorithm at branching points is shown in Algorithm 1. The input of this algorithm is: a de Bruijn graph *G*, the unaligned part of the read *s* and final node *v* of the seed. The goal of this algorithm is to find a path in *G* with the highest similarity score among all possible paths in the graph starting from *v* to the input string *s*. Without loss of generality, consider the case of a seed extension to the right.

The algorithm always considers nodes with a higher priority score first. *PQ* denotes a priority queue whose elements are paths from the root *v*. The priority of an element is the similarity score of that element to (a prefix of) *s*. The algorithm keeps extending a path until a full alignment with *s* is obtained. *bestPath* is then updated with *currPath* if the current path has a higher similarity to *s* than *bestPath*. The algorithm terminates when there are no items left in the queue.


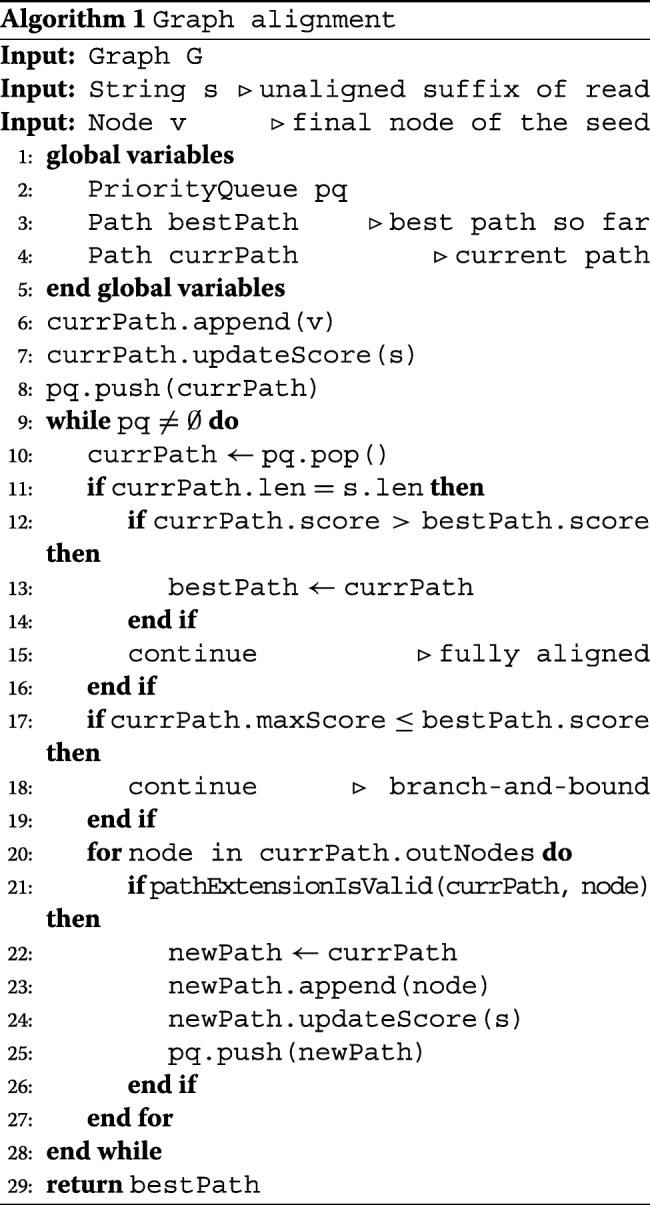


For *w* a path in *G*, *s* a sequence of size *l*, let *f*(*w*,*s*) be the alignment score between *w* and a prefix of *s*, let *f*_*id*_(*s*) be the alignment score of *s* to itself, and let *f*_*max*_(*w*,*s*) be the maximal similarity score between any path in the graph *G* that starts with *w*, and *s*. In our case, *f*_*id*_(*s*)=*ml*, where *m* is the score for a match. Then, given an alignment between a path *w* in *G* and a prefix *s*[1:*n*] of *s*:

$$\begin{aligned} f_{max}(w, s) &\leq f(w, s[1:n]) + f_{id}(s[n+1:l])\\ &= f(w, s[1:n]) + m (l - n). \end{aligned} $$

This bound is used to a priori discard subtrees in *G* in which no path exists with a score that is higher than the best complete alignment found so far. The greedy heuristic of prioritizing extension of the highest scoring paths, combined with this branch and bound strategy narrows down the search space, while still resulting in the optimal alignment between *G* and *s*.

### Implicit repeat resolution using a Markov model

Even though all subsequences of the reference genome can be represented as a contiguous path in the de Bruijn graph, the opposite is not true. In particular, not all paths in the graph that span 3 or more nodes correspond to a subsequence of the reference genome. When extending a path in the alignment process of an individual read, a validation is performed, as shown in Algorithm 1 (line 21). This validation relies on a higher-order (≥ 2) Markov model (MM) and allows skipping paths in the graph that do not occur in the genome. At each branching point, we take into account the topology of the graph and implicitly perform a consensus alignment between all the reads from that genomic region.

To do this, it is necessary to train the model by aligning all reads to the graph and clustering them by genomic region to which they align. However, the model needs to take into account that the data has sequencing errors and the read coverage is not uniform across the genome. The presence of sequencing errors in reads may result in a wrong alignment of reads to the graph. Therefore, the model needs to distinguish between spurious paths which appear to exist in the reference genome because of misaligned reads and true paths. On the other hand, a true path for which the corresponding sequence exists in the genome might not be observed due to a lack of coverage. The probability of observing a true path in the data is smaller for longer paths. The following section describes how BrownieAligner implicitly resolves repeats and guides the aligner using a higher-order MM.

Markov models have been used as a robust statistical framework to model real-time processes in marketing, text analysis, bioinformatics, network analysis, weather forecasting, etc. [15]. One of these applications commonly used in text editors is word prediction. It predicts the most likely word of the user by considering the previously typed words based upon a model that is trained on a broad set of training data [16]. Similarly, in the graph alignment of an individual read, by looking at previously visited nodes, and the information of other reads, the aligner predicts the true path among all possible paths, and prevents alignment against false paths that do not exist in the reference genome.

Formally, an *n*-order Markov Model (*n*-MM) in the de Bruijn graph *G* is defined by:

- A set of states *S*={*s*_1_,…,*s*_*m*_}, in which each state represents a path of *n* nodes (*v*_1_,…,*v*_*n*_) in *G*.
- A set of transition probabilities *p*_*ij*_ representing the probability of extending state path *s*_*i*_ with node *v*_*j*_.

**Fig. 1:**
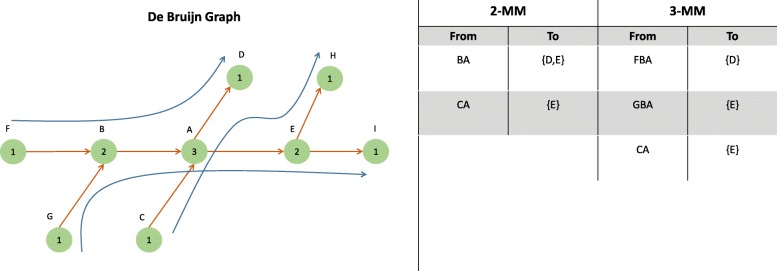
This figure shows the association between the de Bruijn graph and MM tables. On the left side, part of a de Bruijn graph is shown. True paths are depicted by blue lines. The numbers inside each node indicate the multiplicity of that node, i.e., the number of times the node’s sequence is present in the reference genome. A table at each node guides the aligner based on previously observed nodes. The 2-MM and 3-MM tables of node A are shown on the right side. Based on the 2-MM table, reads that align to CA are guided to E as the continuation to node D is not allowed. However, the information in this table is insufficient to guide reads that align to BA since continuations to E and D are both valid. In contrast, the 3-MM table guides the reads that align to FBA to D, and GBA to E. The information in the final row in 3-MM table is redundant because it is also contained in the lower-order 2-MM table

The transition probability between a state path and a node is used to specify whether the path of length *n*+1 exists in the reference genome. We are therefore not particularly interested in the actual values of the transition probabilities; we are merely interested in distinguishing the transitions that do *not* occur (*p*_*ij*_=0) from those that *do* occur (*p*_*ij*_>0). Transitions with zero probability correspond to paths that span *n*+1 nodes in the graph that do not represent a true sequence in the reference genome and can hence be skipped during the read alignment step. The first-order MM is memoryless in that sense that that the prediction of the next node only depends on the current node. Any edge in de Bruijn graph represents a valid overlap between two *k*-mers in the reference genome. Thus, a 1-MM is not informative in this regard. As shown in Fig. 1, the higher-order MM tables can be useful to guide the alignment procedure at branching nodes.

To derive a *n*-MM table, reads are aligned to the graph in a first alignment pass using Algorithm 1 but without any restrictions on alignment path, i.e., without the if-statement in line 21. The goal of this alignment pass is merely to train the MM. Aligned reads imply paths in the graph and all observed paths or subpaths of length *n*+1 are used to populate the MM table. The first *n* nodes of such path define a state *s* while the final *head* node denotes a possible continuation. The table consists of all observed states *s* and the corresponding frequencies of all observed continuations. These frequencies are then converted to probabilities.

However, there can be two types of errors in this process: (1) observing an invalid alignment path because of a misalignment (due to sequencing errors), and (2) missing valid alignment paths because of a lack of coverage.

To minimize the first type of error, we test for each observed path whether its frequency *freq* corresponds to the expected frequency using the following two hypotheses:

- *H*_0_ The multiplicity of the path is zero
- *H*_1_ The multiplicity of the path is at least one

The multiplicity of a path in the graph indicates the number of times that the corresponding sequence appears in the reference genome. Two Poisson distributions are used to model the frequency of observed paths with multiplicity zero (*λ*=1) and multiplicity one (*λ*=*C*_*M*_). Paths for which $likelihoodRatio = \frac {P(freq | H_{0})}{P(freq | H_{1})} \geq minLikelihoodRatio$ are pruned from the list of eligible paths. Here, *likelihoodRatio* is a measure of the degree of certainty of the decision of eliminating a path from the eligible set. The higher this value, the higher the certainty. However, setting a too high value for *minLikelihoodRatio* reduces the ability of the model to avoid false paths.

To minimize the second type of error, the Markov model is only used for paths for which the expected number of reads covering this path *C*_*M*_≥*minChainCov*. Here, *minChainCov* is a second user-defined threshold. Higher values of this parameter reduce the risk of making the second type of error, but again, a too high value reduces the applicability of the Markov model. Given the sequencing coverage *c*, the read length *l*, and a path *P* that implies a sequence of length *M* and multiplicity 1 in the reference genome, the expected coverage *C*_*M*_ of *P* is then given by the following formula:

1 $$  C_{M}= \frac{l-M+1}{l} c  $$

Proof: First, consider a read covering a path of sequence size *M*−1. Second, extend this path with one base, without loss of generality, to the left. For the read to cover this extended path, its start position has to be strictly before the start of the original path. The probability of this is $\frac {l - M + 1}{l - M + 2}$. Hence the following recurrence relation holds:

$$C_{M} = \frac{l-M+1}{l-M+2}C_{M-1}. $$

Solving the recurrence relation leads to

$$C_{M} = \frac{l-M+1}{l}C_{1}. $$

Additionally, *C*_1_=*c* by definition. This concludes the proof.

After constructing the MM tables for different orders as outline before, all reads are again aligned to the graph in a second alignment pass, this time guided by the MM. Different orders are required because a low order might not be sufficiently informative to guide the read alignment whereas a high order might not attain the coverage requirements. In this second alignment pass, the alignment of a read no longer solely depends on the identity between the read and the sequence implied by the alignment path. Rather, the collective information of all other reads is used to identify the true paths in the graph and thus obtain a higher alignment accuracy.

### Choice of parameters:

The scoring system in Algorithm 1 has match, mismatch and gap scores of respectively + 1, − 1 and − 3. The maximum MM order (*maxOrder*) is 10. The values of *minLikelihoodRatio* and *minChainCov* are set respectively to 10^5^ and 10.

### Graph aligner tools

The performance of BrownieAligner is compared with the state-of-the-art graph aligners BGREAT and deBGA. A de Bruijn graph is first constructed from the reference genome, followed by the alignment of reads to the corresponding graph. BrownieAligner and deBGA have the functionality to construct the de Bruijn graph. BGREAT does not support this feature, therefore we used BCALM [17] to construct the de Bruijn graph for BGREAT. A drawback of deBGA is that it only accepts a reference genome as an input and not a graph in general. Therefore, it cannot be used as a graph aligner tool to align reads against the assembly graph. BGREAT and BrownieAligner report corrected reads, i.e., the corresponding sequences from the reference genome after aligning reads, in the same order and file format as the input reads. In contrast, deBGA returns the alignment results in SAM format. Therefore, we developed sam2Alignment script, which is used to produce the corrected read from the reference genome based on the SAM entry. Three versions of BrownieAligner are provided and evaluated in this paper. BrownieAligner is the main tool and benefits from both (1) the greedy branch and bound algorithm and (2) MM repeat resolution. The first feature is disabled in BrownieAlignerNoBB and the second one is disabled in BrownieAlignerNoMM. For all results the default or recommended *k*-mer sizes are used. Parameters and settings are provided in Additional file 1: Data 1.

All tools were run on a machine with four Intel(R) Xeon(R) E5-2698 v3 @ 2.30 GHz CPUs (64 cores in total) and 256 GB of memory. All tools support multithreading and run with 32 threads. Elapsed (wall clock) time and peak resident memory were measured with the GNU *time* command.

### Data

The performance of the three tools was measured on six artificial datasets (see Table 1). For three high-quality reference genomes (*E. coli str. K-12 substr. DH10B*, *Human chr-21* and *Drosophila melanogaster*), reads were simulated for two different Illumina platforms (HiSeq 2000 (100 bp), HiSeq 2500 (150 bp)) using ART [18].

**Table 1:** Artificial datasets used for the evaluation of graph aligner tools

Abbr.	Organism	Reference ID	Genome	Repeated	Sequencing	Cov.	Read
			size	31-mers (%)	platform		length
S1	*Escherichia coli K-12 DH10B*	NC010473	4.5 Mbp	3.2	Illumina HiSeq 2500	25	150 bp
S2	*Escherichia coli K-12 DH10B*	NC010473	4.5 Mbp	3.2	Illumina HiSeq 2000	50	100 bp
S3	*Homo sapiens* Chr. 21	HG19	45.2 Mbp	4.3	Illumina HiSeq 2500	25	150 bp
S4	*Homo sapiens* Chr. 21	HG19	45.2 Mbp	4.3	Illumina HiSeq 2000	50	100 bp
S5	*Drosophila melanogaster*	Release 5	116.4 Mbp	1.1	Illumina HiSeq 2500	25	150 bp
S6	*Drosophila melanogaster*	Release 5	116.4 Mbp	1.1	Illumina HiSeq 2000	50	100 bp

Additionally, the three tools were evaluated on eight real Illumina datasets for which both a reference genome and sequencing data are publicly available (see Table 2). Genome sizes range from 2 Mbp (*Bifidobacterium dentium*) to 116 Mbp (*Drosophila melanogaster*), and read coverage varies from 29 X to 612 X. The data were produced on the Illumina HiSeq, MiSeq and GAII platforms. Read lengths range from 100 bp to 251 bp. Two data sets have a variable read length due to prior read trimming, while the others have fixed read lengths.

**Table 2:** Real datasets used for the evaluation of graph aligner tools

Abbr.	Organism	Reference ID	Genome	Repeated	Cov.	Sequencing	Read	Trimmed	Dataset ID
			size	31-mers (%)		platform	length	reads	
R1	*Bifidobacterium dentium*	Nc013714.1	2.6 Mbp	0.4	373 X	Illumina MiSeq	251 bp		SRR1151311
R2	*Escherichia coli K-12 DH10B*	NC010473	4.5 Mbp	3.2	418 X	Illumina MiSeq	150 bp		Ill. Data library
R3	*Escherichia coli K-12 MG1655*	NC000913	4.5 Mbp	0.6	612 X	Illumina GAII	100 bp		ERA000206
R4	*Salmonella enterica*	NC011083.1	4.7 Mbp	0.5	97 X	Illumina MiSeq	239 bp	✓	SRR1206093
R5	*Pseudomonas aeruginosa*	ERR330008	6.1 Mbp	0.6	169 X	Illumina MiSeq	120 bp	✓	ERR330008
R6	*Homo sapiens* Chr. 21	HG19	45.2 Mbp	4.3	29 X	Illumina HiSeq	100 bp		Ill. Data library
R7	*Caenorhabditis elegans*	WS222	97.6 Mbp	2.6	58 X	Illumina HiSeq	101 bp		SRR543736
R8	*Drosophila melanogaster*	Release 5	116.4 Mbp	1.1	52 X	Illumina HiSeq	100 bp		SRR823377

### Evaluation metrics

For each simulated read, ART generates a corresponding error-free read that is used to perform the accuracy evaluation (see Additional file 1: Data 2). For real data, the ground truth is unknown. In this case, it is assumed that the correct alignment is represented by the alignment of the read to the linear reference genome using BWA. Only paired-end reads where both pairs map to the reference genome properly are extracted using SAMtools [19]. Finally, the pairwise alignment of each read is reconstructed based on the CIGAR string and MD tag using sam2pairwise [20] (see Additional file 1: Data 3). The performance of the aligners is measured based on their ability to align reads to the correct position in the graph. For a given read, the correct path in the graph is the path with the same sequence content as the error-free read corresponding to that read. A detailed explanation is provided in Additional file 1: Data 4.1.

## Results and discussion

### Alignment ratio

Table 3 shows the percentage of correctly aligned reads for the simulated data (see Additional file 1: Data 5.1 for the detailed information). BrownieAligner has the highest percentage of correctly aligned reads (≥ 98.07*%*) for all data sets. BGREAT consistently performs slightly worse (≥ 96.16*%*) than BrownieAligner while deBGA performs slightly worse on half of the data sets (S1, S3, S5), but significantly worse (≥ 83.01*%*) on the others (S2, S4, S6). All tools perform worse on the H. *sapiens* data (S3 and S4) than on the E. *coli* data (S1, S2) and D. *melanogaster* data (S5, S6). The performance of deBGA additionally depends on the read length or coverage, since it consistently performs significantly better on the 150 bp 25x coverage data than on the 100 bp 50x coverage data, for all genomes. Additionally, comparing the results for BrownieAligner and BrownieAlignerNoMM reveals that the use of the Markov model in the read alignment process always improves the overall accuracy of the alignment.

**Table 3:** Accuracy comparison of graph aligner tools in terms of correct alignment of reads to the graph on simulated data

	S1	S2	S3	S4	S5	S6
	Percentage of correctly aligned reads.(%)					
BGREAT	99.94	99.61	98.92	96.16	99.89	99.40
BrownieAligner	100.00	99.99	99.42	98.07	99.97	99.89
BrownieAlignerNoMM	99.99	99.98	99.30	97.67	99.96	99.85
deBGA	99.52	83.48	99.07	83.01	99.37	83.37

We additionally investigated the accuracy of BrownieAligner on those reads that are aligned to a walk in the graph that comprises multiple nodes, i.e., the reads for which the Markov model algorithm is actually used. Table 4 shows the percentage of these reads that are correctly aligned by BrownieAligner (with Markov models) and BrownieAlignerNoMM. Results indicate that the use of these Markov models offers a significant improvement for the alignment of these harder to align reads.

**Table 4:** Accuracy evaluation of BrownieAlignerNoMM and BrownieAligner on the subset of the simulated reads that align to a path of at least two nodes in the graph

	S1	S2	S3	S4	S5	S6
	Percentage of correctly aligned reads. (%)					
BrownieAligner	99.34	99.05	90.72	86.07	98.21	97.12
BrownieAlignerNoMM	98.72	98.47	87.68	82.39	97.38	96.13

In order to see the effect of *k*-mer size on the accuracy, all tools were benchmarked with different values of *k* on all the simulated datasets. The results indicate that for each dataset the best accuracy for BrownieAligner is always higher than the best accuracy for other tools (see Additional file 1: Data 5.1). The results show BrownieAligner performs better for larger *k*. This has two reasons. First, BrownieAligner can use maximal exact matches during the seeding phase, enabling the identification of seeds smaller than *k*. Hence, the sensitivity of the seed finding procedure is not negatively affected by a larger value of *k*. Second, with higher values of *k* the repeat structure in the graph is less complex, and hence BrownieAligner is less prone to choosing an incorrect path in the alignment phase. The accuracy of BrownieAligner on simulated data also has been evaluated based on other values of *maxOrder*, *minLikelihoodRatio* and *minChainCov*. The results indicate that BrownieAligner performs consistently well over a wide range of parameters setting (see Additional file 1: Data 5.1).

Table 5 shows the percentage of reads that are correctly aligned by each tool for 8 real datasets (see Additional file 1: Data 5.2 for the detailed tables). The accuracy of BrownieAligner for the bacterial genomes (R1-R5) is very high (*G*≥99.02*%*) and BrownieAligner outperforms the other tools, followed by deBGA (*G*≥92.36*%*) and then BGREAT (*G*≥84.97*%*). For the H. *sapiens* data (R6) deBGA performs remarkably well. For the other two eukaryotic genomes (R7 and R8), BrownieAligner has again the highest percentage correctly aligned reads. The comparison between BrownieAligner and BrownieAlignerNoMM again indicates that the use of the Markov models to resolve repeats improves the accuracy of read alignment. Additionally, the difference is more significant in H. *sapiens* (R6), which is known to be repeat-rich. The effect of the MM for the alignment of reads that span multiple nodes is further investigated in real data (see Additional file 1: Data 5.2). Results indicate that the alignment accuracy generally benefits from using the MM.

**Table 5:** Accuracy comparison of graph aligner tools in terms of correct alignment of reads to the graph on real data

	R1	R2	R3	R4	R5	R6	R7	R8
	Percentage of correctly aligned reads. (%)							
BGREAT	94.55	94.28	91.28	84.97	96.09	92.01	94.57	80.37
BrownieAligner	99.81	99.81	99.55	99.02	99.78	96.98	96.53	89.59
BrownieAlignerNoMM	99.81	99.80	99.52	98.99	99.78	96.67	96.47	89.55
deBGA	99.67	99.30	92.36	97.31	93.63	98.42	74.72	85.42

### Time and space requirements

**Fig. 2:**
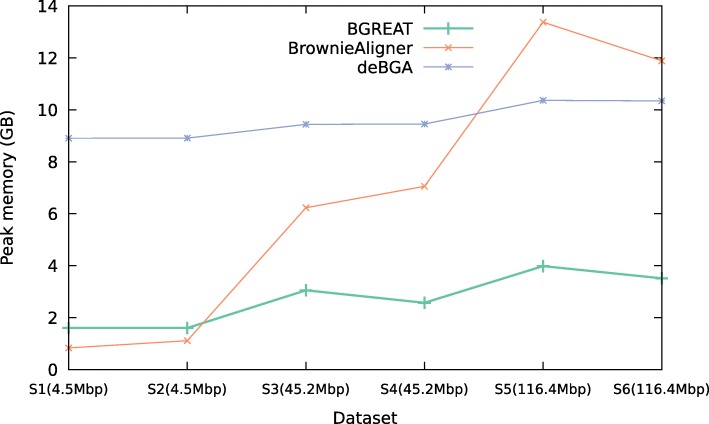
Peak memory usage. Peak memory usage of the aligner tools for simulated datasets

**Fig. 3:**
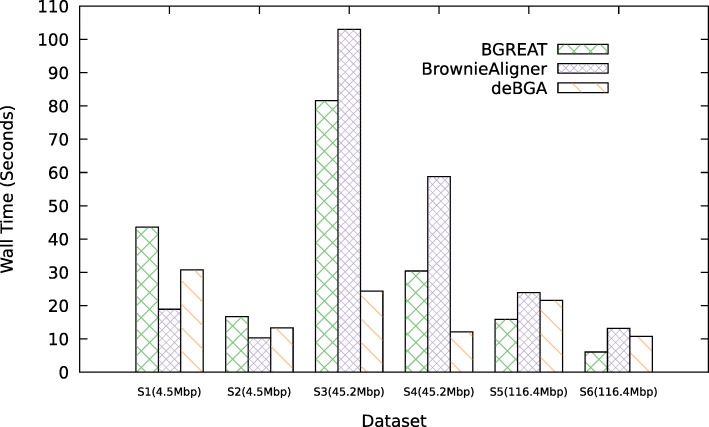
Runtime. Average runtime of tools to align 1M reads for the simulated datasets

Figures 2 and 3 show the memory usage and runtime of the aligners for the simulated data (see Additional file 1: Data 5.3.1 for detailed tables). For the smallest genomes, deBGA requires the most memory, while for larger genomes BrownieAligner has the highest memory requirements. Run times for S1, S2, S5 and S6 data sets are comparable for all tools. However, BrownieAligner and BGREAT take significantly longer than deBGA to align S3 and S4. Generally speaking, BGREAT is memory-efficient and deBGA is fast.

**Fig. 4:**
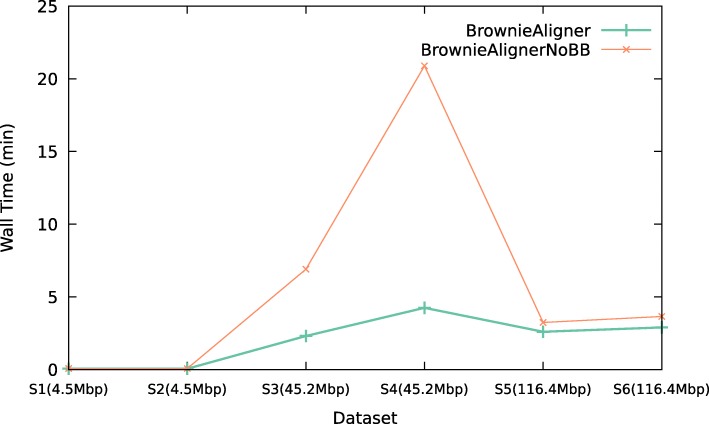
Runtime. The effect of branch and bound strategy on the running time of BrownieAligner

In order to capture the effect of the branch and bound pruning strategy in Algorithm 1, we disabled this feature in BrownieAlignerNoBB. Figure 4 compares the amount of time that the two versions of BrownieAligner take to align only those reads that align to a non-trivial walk in the graph, i.e., those reads where Algorithm 1 is used. Results show that using this strategy reduces the runtime of BrownieAligner especially for more repetitive genomes (see Additional file 1: Data 5.3.1 for detailed tables).

**Fig. 5:**
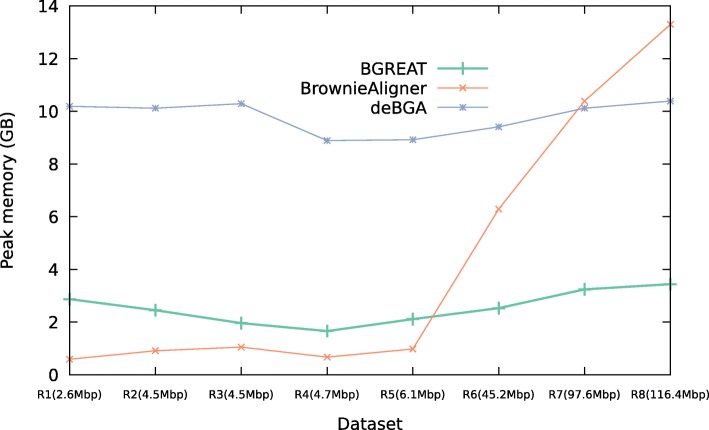
Peak memory usage. Peak memory usage of the aligner tools for real datasets

**Fig. 6:**
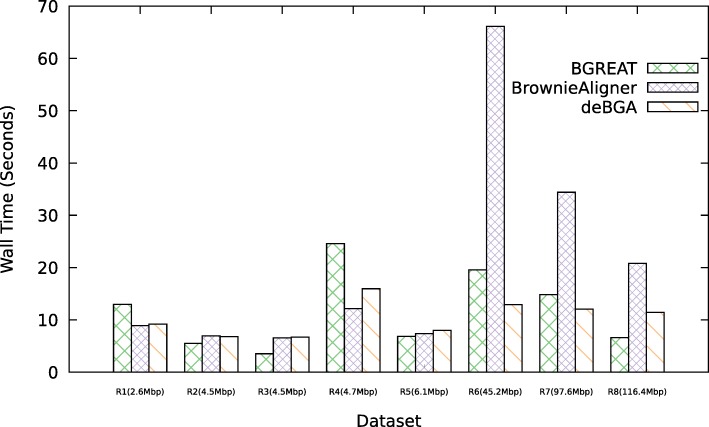
Runtime. Average runtime of tools to align 1M reads for the real datasets

Figures 5 and 6 show the memory usage and runtime of the aligners for the real data (see Additional file 1: Data 5.3.2 for detailed tables). Memory usage and runtime of tools in real data also follow the same pattern as the simulated data except that BrownieAligner is the slowest tool for three largest datasets.

Generally, BrownieAligner has a higher runtime to align reads to the H. *sapiens* genome (S3, S4 in simulated data sets and R6 in real data sets). This is due to the presence of more repetitive patterns in the genome making the de Bruijn graph more complex. Therefore, the DFS algorithm in BrownieAligner has to visit more nodes before it finds the optimal path in the graph.

## Conclusions

BrownieAligner is proposed as a tool to align short Illumina reads to a de Bruijn graph. It uses higher-order Markov models to implicitly resolve repeats in the graph, thus avoiding reads to be aligned against paths in the de Bruijn graph that do not constitute a subsequence of the genome. Our results show that using this model always improves the accuracy of the alignment both in simulated and real data. BrownieAligner generally outperforms other state-of-the-art tools in terms of accuracy, while having similar runtime and memory requirements.

## Additional file

Additional file 1 Supplementary Data: BrownieAligner: Accurate Alignment of Illumina Sequencing Data to de Bruijn Graphs (PDF 166 kb)

## Abbreviations

bp: Base pair
DFS: Depth-first search
GB: Gigabyte
GHz: Gigahertz
Mbp: Megabase pair
MEMs: Maximal exact matches
MM: Markov model
S1…S6: Simulated dataset 1…6
R1…R8: Real dataset 1…8
