# Supplementary material for: BrownieAligner: accurate alignment of Illumina sequencing data to de Bruijn graphs
Source: BMC Bioinformatics. 2018 Sep 4;19:311. doi: 10.1186/s12859-018-2319-7 (PMC6122196; doi:10.1186/s12859-018-2319-7)
Supplement: Supplementary file 1 — Supplementary Data: BrownieAligner: Accurate Alignment of Illumina Sequencing Data to de Bruijn Graphs (PDF 166 kb) [file 12859_2018_2319_MOESM1_ESM.pdf]

BrownieAligner: Accurate Alignment of Illumina Sequencing Data to de Bruijn Graphs

Mahdi Heydari[1,2], Giles Miclotte[1,2],
Yves Van de Peer[2,3,4,5], and Jan Fostier[1,2]

[1]Department of Information Technology, Ghent University-imec, IDLab, Ghent, Belgium
[2]Bioinformatics Institute Ghent, Ghent, Belgium
[3]Center for Plant Systems Biology, VIB, Ghent, Belgium
[4]Department of Plant Biotechnology and Bioinformatics, Ghent University, Ghent, Belgium
[5]Department of Genetics, Genome Research Institute, University of Pretoria, Pretoria, South Africa

Contact: jan.fostier@ugent.be

Contents

1 Parameter Settings **3**
1.1 BGREAT . . . 3
1.2 BrownieAligner . . . 3
1.3 deBGA . . . 3

2 Simulated data preparation **4**

3 Real data preparation **4**

4 Evaluation Metric **4**
4.1 Alignment ratio . . . 4

5 Results **6**
5.1 Simulated Data . . . 6
5.1.1 BGREAT . . . 7
5.1.2 BrownieAligner . . . 7
5.1.3 deBGA . . . 7
5.1.4 Choice of parameters . . . 8
5.2 Real Data . . . 8
5.3 Time and space requirements . . . 10
5.3.1 Simulated data . . . 10
5.3.2 Real data . . . 11

1 Parameter Settings

All tools were executed with 32 threads. For all tables and figures in the main paper and in the supplementary data the default or recommended values of parameters were taken for each tool. Below, the command line parameters are specified for each tool individually:

1.1 BGREAT[1]

BCALM is used to build the de Bruijn graph from the reference genome.

```
$ ./bcalm -nb-cores 32 -in genome.fasta -kmer-size 31 -abundance-min 1
$ ./bgreat -c -q -O -u $inputreads -g genome.unitigs.fa -k 31 -t 32
```

1.2 BrownieAligner[2]

```
$ ./brownie index -t 32 -p $outputDir -k 31 genome.fasta
$ ./brownie align -t 32 -p $outputDir -k 31 -o $outputDir/outputFile $inputreads
```

To disable branch and bound in the alignment procedure:

```
$ ./brownie align -nBB -t 32 -p $outputDir -k 31 -o $outputDir/outputFile
$inputreads
```

To disable Markov Model in the alignment procedure:

```
$ ./brownie align -nMM -t 32 -p $outputDir -k 31 -o $outputDir/outputFile
$inputreads
```

1.3 deBGA v. 0.1[3]

deBGA initially builds the graph from the reference genome. Then, it aligns reads to the graph and returns the result as a SAM file. sam2Alignment then constructs the corrected read from the reference genome based on the corresponding alignment position and the cigar string in the SAM file. deBGA sometimes reports multiple alignments for one read. In this case only the one with the lowest edit distance is considered. In order to measure the runtime and the memory usage of deBGA only two first steps (deBGA index, and deBGA aln) are taken into consideration.

```
$ ./deBGA index genome.fasta reference/ -p 32
$ ./deBGA aln reference/ $inputreads1 $inputreads2 deBGA.sam -p 32
$ ./sam2Alignment deBGA.sam genome.fasta $inputreads SMID
```

[1] https://github.com/Malfoy/BGREAT2.git
[2] https://github.com/biointec/browniealigner
[3] https://github.com/hitbc/deBGA.git

2 Simulated data preparation

Synthetic Illumina reads from two different Illumina platforms and in two different read lengths and coverage (HiSeq 2000 (100 bp and 50X), HiSeq 2500 (150 bp and 25X)) are generated with ART read simulator (v. 2.6). The following commands were used:

```
$./art_illumina -ss HS20 -sam -i genome.fasta -p -l 100 -f 50 -m 200 -s 10
-o reads
$./art_illumina -ss HS25 -sam -i genome.fasta -p -l 150 -f 25 -m 200 -s 10
-o reads
```

The mean fragment size is 200 bp, and the fragment standard deviation is 10 bp.

3 Real data preparation

In the absence of ground truth for real data, it is assumed that the error-free read is represented by the segment of the reference genome to which that read aligns. Therefore, reads are initially aligned to the linear reference genome by BWA. Then paired-end reads that both pairs map to the reference genome properly are extracted and stored into mappedPairs.sam file. sam2pairwise tool uses the CIGAR and MD tag to reconstruct the pairwise alignment of each read. Finally, the python script is used to extract the mappedReads (uncorrected mapped reads), perfectReads (equivalent error free reads) from the pairwise alignment and initial real data.

```
$ bwa index reference/genome.fasta
$ bwa mem reference/genome.fasta -t 16 reads.fastq -p >bwa.sam
$ samtools view -S -f 0x2 -F 0x904 bwa.sam > mappedPairs.sam
$ sam2pairwise <mappedPairs.sam> ali.alignment
$ python extMappedFromAli.py reads.fastq ali.alignment perfectReads.fasta
mappedReads.fastq
```

4 Evaluation Metric

4.1 Alignment ratio

Each tool reports set of reads that are aligned to the graph either explicitly (BGREAT and BrownieAligner in the output) or implicitly (deBGA: by the corresponding flag in the SAM file). However, not all of thees reads align correctly to the graph. Generally, reads are classified into three groups as follows :

1. Aligned reads
   (a) Correctly aligned (CA)
   (b) Incorrectly aligned (IA)
2. Not aligned reads (NA)

While reads that belong to the NA class are specified by each tool, rest of the reads are needed to be further classified into CA and IA classes. The classification is straightforward when the ground

truth, i.e., the perfect read, is known. Let R represent an input read. For each read R, there is a corresponding read C which is the segment of the reference genome to which that read aligns and represented by a path in the graph, and P which is the ground truth (error free read). We define a correct alignment as such P is identical to C. Then, read R is categorized into CA group if the alignment is correct, otherwise into IA group.

5 Results

5.1 Simulated Data

Detailed information about the accuracy of tools on simulated data which includes percentage of correctly aligned reads and total number of (aligned, correctly aligned, incorrectly aligned and unaligned) reads are shown in Table 1. Default k-mer sizes are used for all tools which is 31 in BGREAT and BrownieAligner and 22 in deBGA. Additionally, BGREAT, BrownieAligner and deBGA are benchmarked against all datasets with different k in 5.1.1, 5.1.2 and 5.1.3 sections respectively.

Table 1: Accuracy evaluation of graph aligners on simulated data

	S1 (*E. coli DH10B*)	S2 (*E. coli DH10B*)	S3 (*H. sapiens* Chr. 21)	S4 (*H. sapiens* Chr. 21)	S5 (*D. melanogaster*)	S6 (*D. melanogaster*)
			**Percentage of correctly aligned reads.**			
BGREAT	99.94	99.61	98.92	96.16	99.89	99.40
BrownieAligner	100.00	99.99	99.42	98.07	99.97	99.89
BrownieAlignerNoMM	99.99	99.98	99.30	97.67	99.96	99.85
deBGA	99.52	83.48	99.07	83.01	99.37	83.37
			**Total number of aligned reads.**			
BGREAT	780 568	2 334 082	7 746 106	22 875 994	20 046 023	59 878 851
BrownieAligner	780 982	2 342 838	7 759 283	23 092 285	20 059 808	60 146 026
BrownieAlignerNoMM	780 966	2 342 698	7 750 237	23 010 993	20 057 931	60 128 263
deBGA	777 771	1 958 626	7 748 352	19 836 023	19 970 349	50 355 578
			**Total number of correctly aligned reads.**			
BGREAT	780547	2333855	7701160	22458474	20041869	59832087
BrownieAligner	780968	2342711	7739916	22904206	20057579	60121746
BrownieAlignerNoMM	780951	2342548	7730321	22811819	20055622	60101941
deBGA	777247	1955941	7712571	19387610	19936325	50181809
			**Total number of incorrectly aligned reads.**			
BGREAT	21	227	44 946	417 520	4 154	46 764
BrownieAligner	14	127	19 367	188 079	2 229	24 280
BrownieAlignerNoMM	15	150	19 916	199 174	2 309	26 322
deBGA	524	2 685	35 781	448 413	34 024	173 769
			**Total number of unaligned reads.**			
BGREAT	432	8 968	38 870	478 956	17 505	311 799
BrownieAligner	18	212	25 693	262 665	3 720	44 624
BrownieAlignerNoMM	34	352	34 739	343 957	5 597	62 387
deBGA	3 229	384 424	36 624	3 518 927	93 179	9 835 072

5.1.1 BGREAT

Table 2 shows the accuracy of BGREAT in terms of percentage of correctly aligned reads for different values of k on simulated data. The default value is 31.

Table 2: Accuracy evaluation of BGREAT on simulated data for different values of k.

k-mer	S1 (*E. coli DH10B*)	S2 (*E. coli DH10B*)	S3 (*H. sapiens* Chr. 21)	S4 (*H. sapiens* Chr. 21)	S5 (*D. melanogaster*)	S6 (*D. melanogaster*)
			**Percentage of correctly aligned reads.**			
25	99.94	**99.83**	98.53	95.23	99.86	**99.50**
31	99.94	99.61	98.92	96.16	99.89	99.40
35	99.94	99.44	99.13	96.66	99.91	99.29
41	**99.95**	99.44	99.28	97.18	99.91	99.29
51	**99.95**	99.41	**99.45**	**97.62**	**99.92**	99.25

5.1.2 BrownieAligner

Table 3 shows the accuracy of BrownieAligner in terms of percentage of correctly aligned reads for different values of k on simulated data. The default value is 31. Larger sizes of k

Table 3: Accuracy evaluation of BrownieAligner on simulated data for different values of k.

k-mer	S1 (*E. coli DH10B*)	S2 (*E. coli DH10B*)	S3 (*H. sapiens* Chr. 21)	S4 (*H. sapiens* Chr. 21)	S5 (*D. melanogaster*)	S6 (*D. melanogaster*)
			**Percentage of correctly aligned reads.**			
25	100.0	99.98	99.23	97.46	99.96	99.86
31	100.0	**99.99**	99.42	98.07	99.97	99.89
35	100.0	**99.99**	99.49	98.31	99.97	99.90
41	100.0	**99.99**	99.57	98.53	**99.98**	99.90
51	100.0	**99.99**	**99.66**	**98.74**	**99.98**	**99.91**

5.1.3 deBGA

Table 4 shows the accuracy of deBGA for different values of k in terms of percentage of correctly aligned reads on simulated data. The default value is 22, and the accepted range is [22,28].

Table 4: Accuracy evaluation of deBGA on simulated data for different values of k.

k-mer	S1 (*E. coli DH10B*)	S2 (*E. coli DH10B*)	S3 (*H. sapiens* Chr. 21)	S4 (*H. sapiens* Chr. 21)	S5 (*D. melanogaster*)	S6 (*D. melanogaster*)
			**Percentage of correctly aligned reads.**			
22	99.52	**83.48**	99.07	83.01	99.37	83.37
24	99.52	83.47	99.09	83.04	99.37	**83.38**
26	99.52	83.40	**99.12**	**85.66**	99.37	83.30
28	99.52	83.34	**99.12**	85.60	**99.38**	83.26

5.1.4 Choice of parameters

The results in the main paper are based on these parameters: the maximum MM order (maxOrder) is 10, the minLikelihoodRatio and minChainCov are set respectively to 10^5 and 10. However, we additionally investigated the accuracy of BrownieAligner on simulated data based on other values of these parameters. Table 5 shows the accuracy of BrownieAligner in terms of percentage of correctly aligned reads for different values of maxOrder. The default value is 10.

Table 5: Accuracy evaluation of BrownieAligner on simulated data for different values of maxOrder.

maxOrder	S1 (*E. coli DH10B*)	S2 (*E. coli DH10B*)	S3 (*H. sapiens* Chr. 21)	S4 (*H. sapiens* Chr. 21)	S5 (*D. melanogaster*)	S6 (*D. melanogaster*)
			**Percentage of correctly aligned reads.**			
5	100.0	99.98	99.37	97.90	99.97	99.88
10	100.0	99.99	99.42	98.07	99.97	99.89
15	100.0	99.99	99.45	98.16	99.97	99.89
20	100.0	99.99	99.46	98.21	99.97	99.89

Table 6 shows the accuracy of BrownieAligner in terms of percentage of correctly aligned reads for different values of minLikelihoodRatio. The default value is 10^5.

Table 6: Accuracy evaluation of BrownieAligner on simulated data for different values of minLikelihoodRatio.

minLikelihoodRatio	S1 (*E. coli DH10B*)	S2 (*E. coli DH10B*)	S3 (*H. sapiens* Chr. 21)	S4 (*H. sapiens* Chr. 21)	S5 (*D. melanogaster*)	S6 (*D. melanogaster*)
			**Percentage of correctly aligned reads.**			
10^2	100.0	99.99	99.43	98.08	99.97	99.89
10^5	100.0	99.99	99.42	98.07	99.97	99.89
10^{10}	99.99	99.98	99.34	98.01	99.96	99.88
10^{15}	99.99	99.98	99.34	97.78	99.96	99.86

Table 7 shows the accuracy of BrownieAligner in terms of percentage of correctly aligned reads for different values of minChainCov. The default value is 10.

Table 7: Accuracy evaluation of BrownieAligner on simulated data for different values of minChainCov.

minChainCov	S1 (*E. coli DH10B*)	S2 (*E. coli DH10B*)	S3 (*H. sapiens* Chr. 21)	S4 (*H. sapiens* Chr. 21)	S5 (*D. melanogaster*)	S6 (*D. melanogaster*)
			**Percentage of correctly aligned reads.**			
5	100.0	99.99	99.42	98.07	99.97	99.89
10	100.0	99.99	99.42	98.07	99.97	99.89
15	100.0	99.99	99.42	98.07	99.97	99.89
20	99.99	99.99	99.41	98.07	99.97	99.88

The results in this section indicates the accuracy of BrownieAligner is not affected by changing the parameters. Generally increasing the maximum order of Markov model can slightly improve the accuracy and increasing minLikelihoodRatio and minChainCov can marginally reduce the accuracy.

5.2 Real Data

Detailed information about the accuracy of tools on real data which includes percentage of correctly aligned reads and total number of (aligned, correctly aligned, incorrectly aligned and unaligned) reads are shown in Table 8.

Table 8: Accuracy comparison of graph aligners on real data

Tool	*B. dentium*	*E. coli DH10B*	*E. coli MG1655*	*S. enterica*	*P. aeruginosa*	*H. sapiens* Chr. 21	*C. elegans*	*D. melanogaster*
				**Percentage of correctly aligned reads.**				
BGREAT	94.55	94.28	91.28	84.97	96.09	92.01	94.57	80.37
BrownieAligner	99.81	99.81	99.55	99.02	99.78	96.98	96.53	89.59
BrownieAlignerNoMM	99.81	99.80	99.52	98.99	99.78	96.67	96.47	89.55
deBGA	99.67	99.3	92.36	97.31	93.63	98.42	74.72	85.42
				**Total number of aligned reads.**				
BGREAT	3 668 594	12 270 256	25 852 298	1 604 703	8 225 144	12 396 108	44 732 411	46 605 426
BrownieAligner	3 873 574	12 991 343	28 199 093	1 870 902	8 542 522	13 115 847	45 639 967	53 372 339
BrownieAlignerNoMM	3 873 499	12 990 034	28 192 797	1 870 430	8 542 199	13 067 671	45 609 800	53 329 978
deBGA	3 873 217	12 967 068	26 275 097	1 852 597	8 032 450	13 305 423	35 970 874	52 372 714
				**Total number of correctly aligned reads.**				
BGREAT	3668445	12268614	25837931	1604075	8224087	12254814	43710499	45520854
BrownieAligner	3872578	12987725	28176839	1869313	8539899	12916306	44616422	50741321
BrownieAlignerNoMM	3872494	12986226	28169892	1868762	8539540	12875329	44590192	50718731
deBGA	3867008	12921137	26141401	1837075	8013318	13109192	34538893	48378269
				**Total number of incorrectly aligned reads.**				
BGREAT	149	1 642	14 367	628	1 057	141 294	1 021 912	1 084 572
BrownieAligner	996	3 618	22 254	1 589	2 623	199 541	1 023 545	2 631 018
BrownieAlignerNoMM	1 005	3 808	22 905	1 668	2 659	192 342	1 019 608	2 611 247
deBGA	6 209	45 931	133 696	15 522	19 132	196 231	1 431 981	3 994 445
				**Total number of unaligned reads.**				
BGREAT	211 338	742 308	2 452 766	283 171	333 374	922 994	1 489 463	10 033 384
BrownieAligner	6 358	21 221	105 971	16 972	15 996	203 255	581 907	3 266 471
BrownieAlignerNoMM	6 433	22 530	112 267	17 444	16 319	251 431	612 074	3 308 832
deBGA	6 715	45 496	2 029 967	35 277	526 068	13 679	10 251 000	4 266 096

The accuracy of BrownieAligner on those reads that are aligned to a walk in the graph that contains multiple unitigs is shown in Table 9 .

Table 9: Accuracy evaluation of BrownieAlignerNoMM and BrownieAligner on the subset of the real data that are corrected by DFS Algorithm.

Tool	*B. dentium*	*E. coli DH10B*	*E. coli MG1655*	*S. enterica*	*P. aeruginosa*	*H. sapiens* Chr. 21	*C. elegans*	*D. melanogaster*
				**Percentage of correctly aligned reads.**				
BrownieAligner	97.83	97.15	94.94	93.75	96.08	67.95	50.70	39.09
BrownieAlignerNoMM	96.81	94.50	92.57	90.14	94.18	63.16	49.18	38.55

5.3 Time and space requirements

5.3.1 Simulated data

The memory usage and run time of the aligners are shown as plots in the paper, Table 10 and Table 11 respectively show the corresponding values. Additionally, a plot showing the effect of the branch and bound algorithm on the run time of BrownieAligner on the simulated data is shown in the paper, the corresponding values are provided in Table 12.

Table 10: Peak memory (GB) usage of the aligners on simulated data

Tools	S1 (*E. coli DH10B*)	S2 (*E. coli DH10B*)	S3 (*H. sapiens* Chr. 21)	S4 (*H. sapiens* Chr. 21)	S5 (*D. melanogaster*)	S6 (*D. melanogaster*)
BGREAT	1.60	1.60	**3.05**	**2.57**	**3.98**	**3.51**
BrownieAligner	**0.83**	**1.11**	6.23	7.05	13.37	11.88
deBGA	8.90	8.91	9.44	9.45	10.36	10.34

Table 11: Run time (min) of the aligners on simulated data

Tools	S1 (*E. coli DH10B*)	S2 (*E. coli DH10B*)	S3 (*H. sapiens* Chr. 21)	S4 (*H. sapiens* Chr. 21)	S5 (*D. melanogaster*)	S6 (*D. melanogaster*)
BGREAT	0.57	0.65	10.59	11.84	**5.31**	**6.08**
BrownieAligner	**0.25**	**0.40**	13.36	22.88	7.99	13.23
deBGA	0.40	0.52	**3.16**	**4.70**	7.21	10.82

Table 12: Effect of the branch and bound strategy on the run time (min) of BrownieAligner on simulated data

Tools	S1 (*E. coli DH10B*)	S2 (*E. coli DH10B*)	S3 (*H. sapiens* Chr. 21)	S4 (*H. sapiens* Chr. 21)	S5 (*D. melanogaster*)	S6 (*D. melanogaster*)
BrownieAligner	**0.06**	**0.06**	**2.31**	**4.25**	**2.60**	**2.90**
BrownieAlignerNoBB	0.07	0.07	6.90	20.89	3.24	3.65

5.3.2 Real data

Table 13 shows the peak memory usage of aligners on real data. Table 14 shows the run time (wall time) of aligners on real data.

Table 13: Peak memory (GB) usage of the aligners on real data.

Tool	*B. dentium*	*E. coli DH10B*	*E. coli MG1655*	*S. enterica*	*P. aeruginosa*	*H. sapiens* Chr. 21	*C. elegans*	*D. melanogaster*
BGREAT	2.87	2.45	1.96	1.66	2.11	**2.53**	**3.24**	**3.44**
BrownieAligner	**0.59**	**0.91**	**1.05**	**0.67**	**0.98**	6.29	10.39	13.30
deBGA	10.19	10.12	10.29	8.89	8.92	9.41	10.12	10.39

Table 14: Run time (min) of the aligners on real data

Tool	*B. dentium*	*E. coli DH10B*	*E. coli MG1655*	*S. enterica*	*P. aeruginosa*	*H. sapiens* Chr. 21	*C. elegans*	*D. melanogaster*
BGREAT	0.84	**1.19**	**1.66**	0.77	**0.98**	4.34	11.42	**6.26**
BrownieAligner	**0.58**	1.51	3.08	**0.38**	1.05	14.69	26.50	19.63
deBGA	0.59	1.47	3.16	0.50	1.14	**2.87**	**9.30**	10.78
